# A case report of dermatofibrosarcoma protuberans with osteolytic

**DOI:** 10.1186/s12891-025-09073-1

**Published:** 2025-08-19

**Authors:** Zuyao Song, Weiyi Gong, Hongyan Zhang, Yuqiang Wang, Wenfei Wang, Xiaokun Hu

**Affiliations:** 1 Shengli Oilfield Central Hospital, No. 31 Jinan Road, Dongying City, Shandong Province China; 2 Affiliated Hospital of Qingdao University, No. 1677, Wutai Mountain Road, Huangdao District, Qingdao City, Shandong Province China

**Keywords:** Bone metastasis, Dermatofibrosarcoma protuberans, CT, MRI

## Abstract

**Background:**

Dermatofibrosarcoma protuberans (DFSP) is a rare, low-to-intermediate grade malignant soft tissue sarcoma that is prone to local recurrence after surgery but rarely metastasizes to distant sites. However, in this case, the tumor metastasized to the humerus and presented as a solitary lesion.

**Case presentation:**

A 42-year-old male patient presented with pain and limited mobility in the left upper limb, with a history of surgery for DFSP in the popliteal fossa. Imaging examinations revealed bone destruction in the proximal humerus and a soft tissue mass, with the lesion measuring 11.0 × 8.5 × 7.0 cm. Aspiration biopsy confirmed that the lesion in the humerus was a metastatic tumor. After two months of palliative treatment, follow-up examinations showed a reduction in the size of the lesion.Seven months after the treatment, the humeral lesion had progressed, and multiple new bone metastases were found.

**Conclusions:**

Metastasis of DFSP is rare, and bone metastasis is even rarer. We need to integrate medical history, perform comprehensive diagnosis through immunohistochemistry, molecular pathology monitoring, and imaging examinations. Additionally, this case reminds us that when encountering patients who primarily complain of bone pain during the initial diagnosis, we should thoroughly inquire about their medical and surgical history, conduct a comprehensive analysis, and make an integrated judgment. When necessary, perform a core biopsy first to determine the origin of the lesion. This approach allows us to provide patients with as comprehensive a range of treatment options as possible, thereby improving their quality of life.

## Background

Dermatofibrosarcoma protuberans(DFSP) is a rare, moderately malignant soft tissue sarcoma that predominantly affects individuals aged 20–50, with a predilection for the trunk, followed by the limbs, face, and neck [1, 2]. It is characterized by slow growth, strong local invasiveness, a tendency to recur after surgery, but a low propensity for distant metastasis [3, 4].The metastasis rate is usually less than 6%, with the lungs being the primary site of metastasis, followed by bones (including the ribs), the brain, and the heart [5].

### Case report

**Fig. 1:**
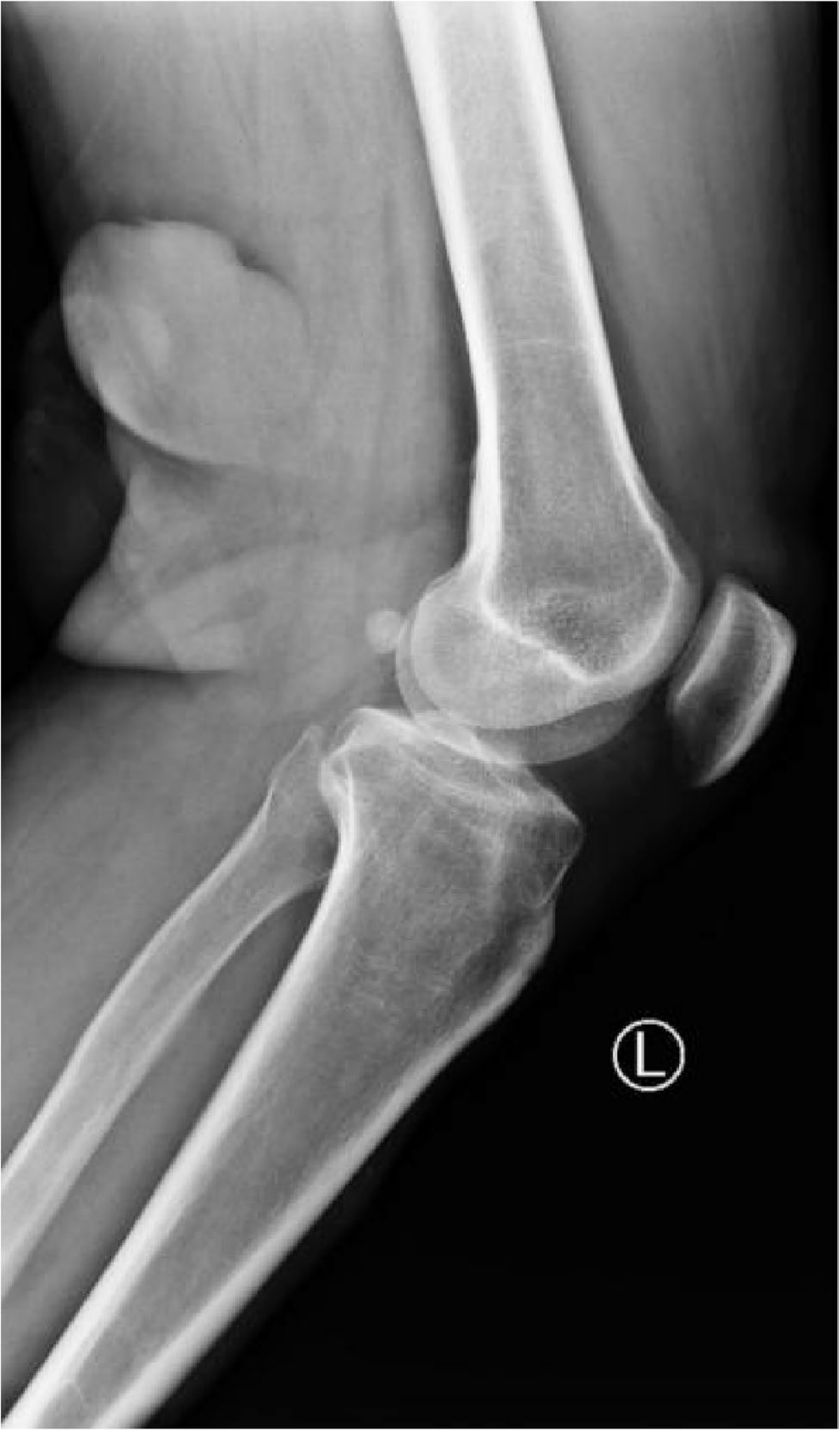
2023.03 X-ray shows a round mass is observed in the left popliteal fossa, with clear boundaries and a distinct separation from the femur. (This refers to the lesion mentioned in the past medical history from 1.5 years ago, which has since been treated with surgery and radiotherapy.)

A 42-year-old male presented with left upper limb pain and limited mobility for half a month without any apparent cause. He underwent rehabilitation treatment at a traditional Chinese medicine hospital, experienced some relief, but later the pain intensified. An MRI of the left shoulder revealed abnormal signals within the left humerus. He has a history of DFSP surgery on the left popliteal fossa 18 months ago (Fig. 1 shows the preoperative X-ray).

**Fig. 2:**
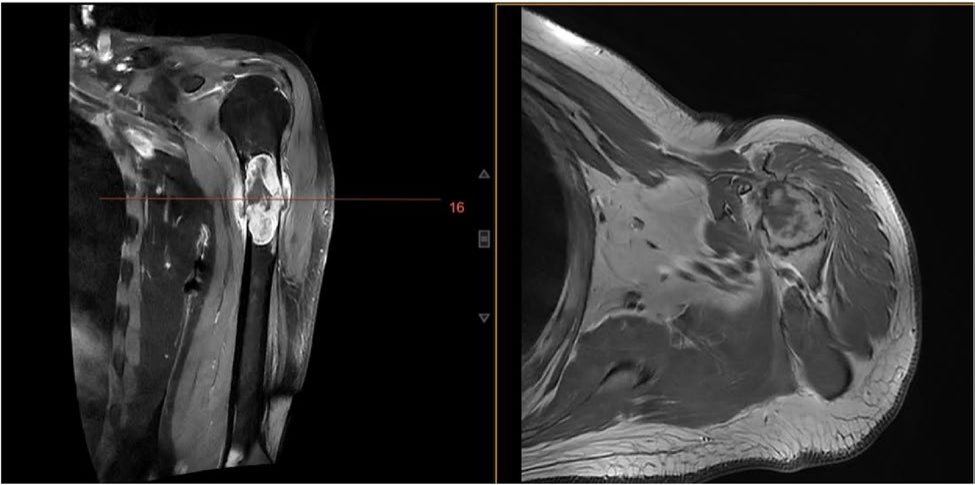
(MRI)The coronal(**L**) and axial(**R**) views show a mass-like abnormal enhancement signal within the upper medullary cavity of the left humerus, with the long axis of the lesion parallel to the long axis of the humerus

2024-08-09 Magnetic Resonance Imaging (MRI) (Fig. 2) shows: In the upper medullary cavity of the left humerus, there is a mass-like abnormal signal with an approximate size of 6.2 cm × 2.4 cm × 2.6 cm. The solid components show significant enhancement on enhanced scanning, with surrounding soft tissues showing patchy significant enhancement.

**Fig. 3:**
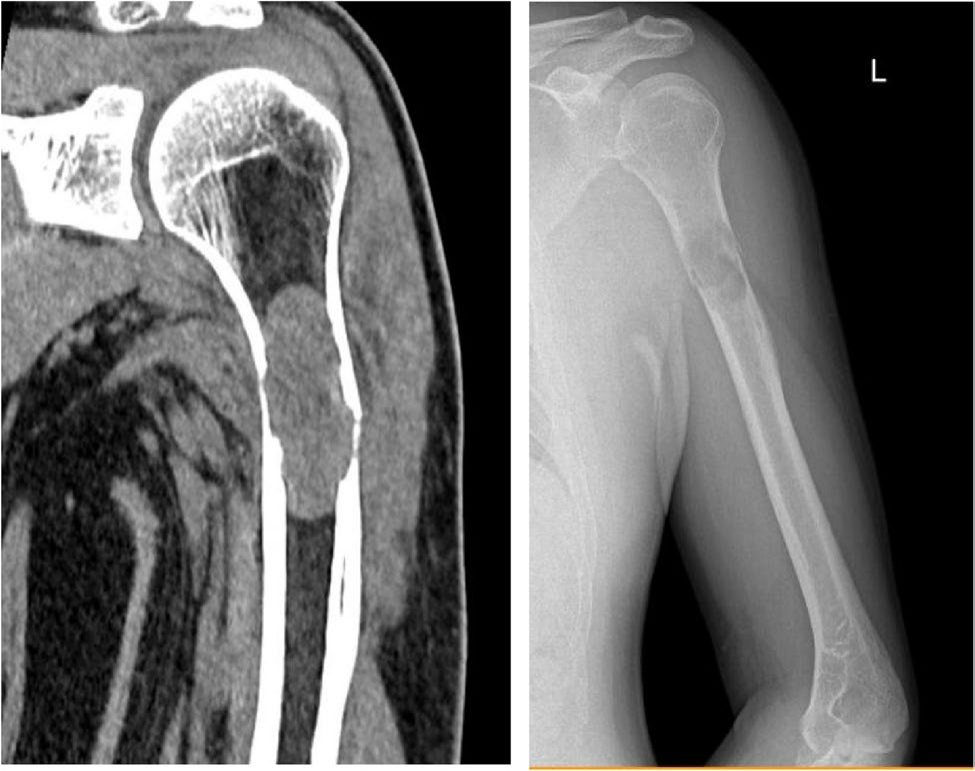
CT(**L**) and X-ray(**R**) scans show the left humerus upper segment shows an elongated oval-shaped low-density shadow, with adjacent cortical thinning and fracture

2024-08-10 Computerized Tomography (CT) scan (Fig. 3) shows: In the upper segment of the left humerus, there is an elongated oval-shaped low-density shadow, approximately 2.8 cm × 2.9 cm × 6.3 cm in size, with a CT value of about 27.9 HU. The long axis is parallel to the long axis of the humerus, with adjacent cortical thinning and local discontinuity. The ends of the break are reasonably well-aligned, and there is surrounding soft tissue swelling.

The patient underwent a popliteal fossa mass excision surgery 18 months ago. The pathology was “Dermatofibrosarcoma Protuberans (Fibrosarcomatous Type),” with a size of approximately 11.0 cm × 8.5 cm × 7.0 cm. The tumor involved the epidermis and formed a superficial ulcer. The upper, lower, inner, outer, and deep surgical margins were all free of tumor. Of the 3 left inguinal lymph nodes examined, none showed evidence of tumor metastasis. Immunohistochemical results were as follows: CD34 (3+), Vimentin (3+), P53 (wild type), CD99 (focally positive), Bcl-2 (+), Ki-67 (+, 15–20%). Three months postoperatively, the patient underwent radiotherapy (specific details not provided).

**Fig. 4:**
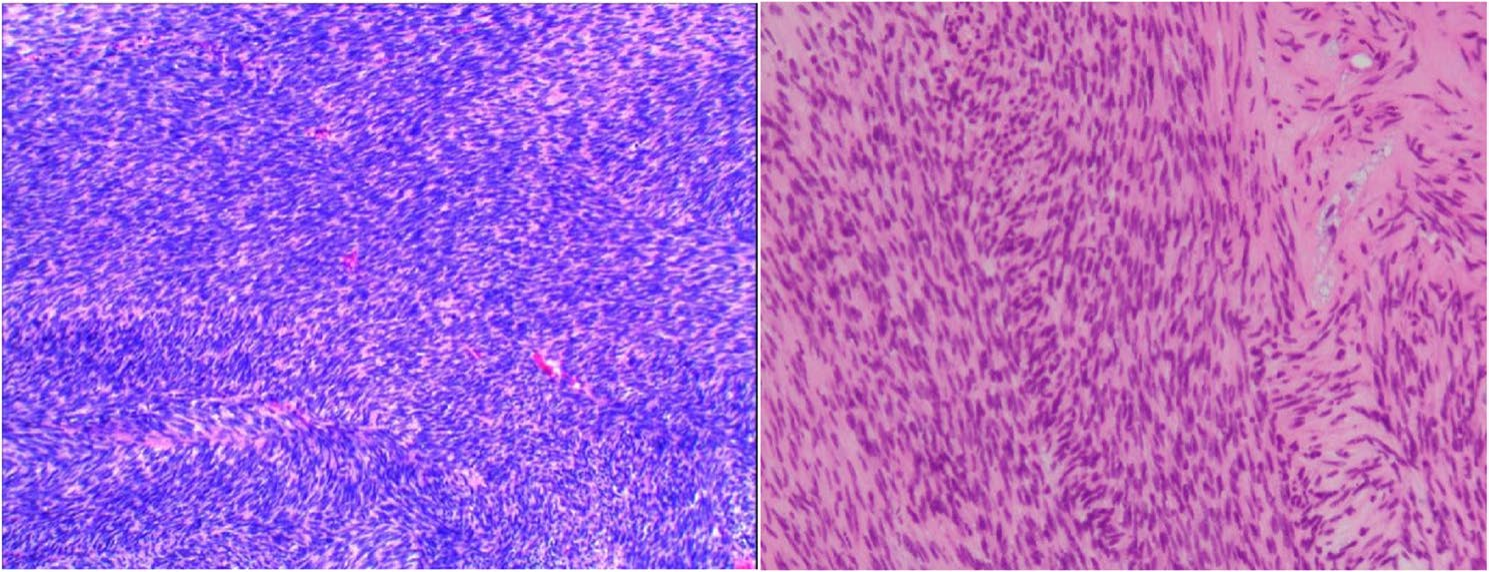
Pathology shows that both the popliteal fossa (**L**) and the humerus (**R**) are Dermatofibrosarcoma Protuberans (Fibrosarcomatous type). The tumor cells are composed of densely packed spindle-shaped cells arranged in a herringbone or fishbone pattern

2024-08-14 A percutaneous core biopsy of the mass within the upper medullary cavity of the left humerus was performed (Fig. 4). Gross observation: Two grayish-white linear tissues (1.0–1.5 cm in length, 0.1 cm in diameter, soft in texture). Pathology revealed Dermatofibrosarcoma Protuberans (Fibrosarcomatous Type). Immunohistochemistry results: Vimentin (3+), CD34 (focally weakly positive), SMA (2+), Actin/MSA (+),Bcl-2(-), CD99(-), Ki-67 (+, 10–13%). A whole-body bone scan was performed, which revealed radiotracer uptake in the left humerus, with no other bones showing obvious abnormal accumulation or deficiency of radioactivity.A multidisciplinary team consultation across the hospital and remote consultation outside the hospital were requested. Based on the biopsy pathology results, history of DFSP in the left popliteal fossa, and the patient’s clinical symptoms, a palliative treatment plan was provided, with Imatinib administered orally at a dose of 400 mg per day.

**Fig. 5:**
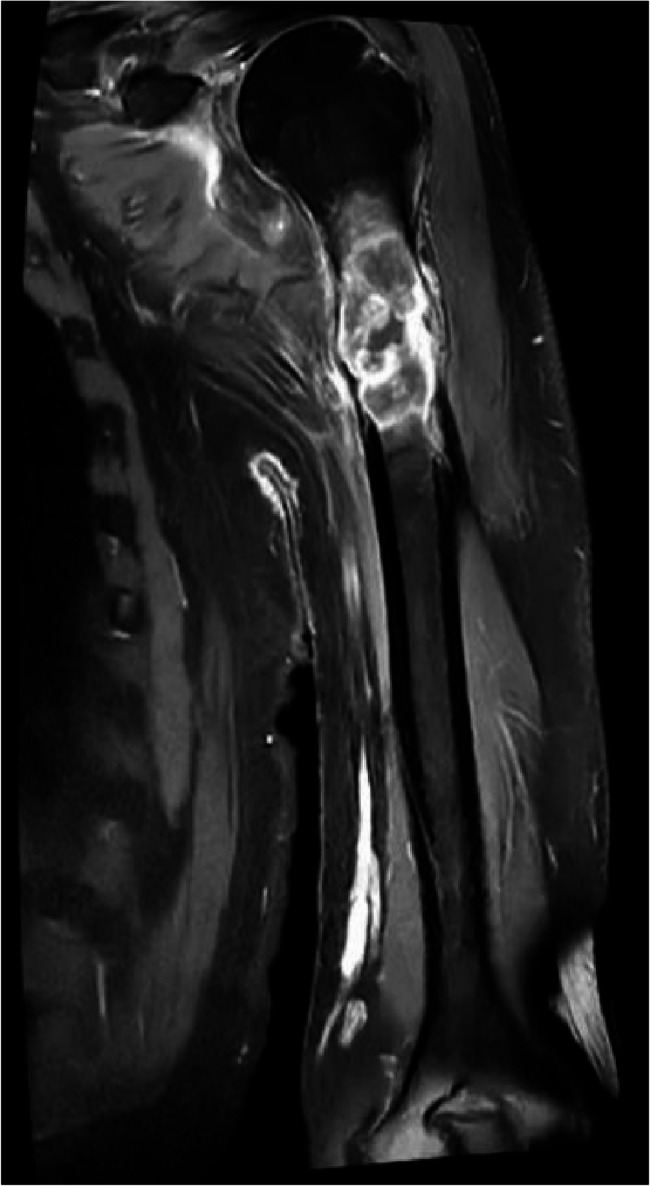
Two months after treatment, follow-up MRI shows: The lesion within the upper medullary cavity of the left humerus has decreased in size compared to before

Two months later, a follow-up MRI scan (Fig. 5) shows: In the upper medullary cavity of the left humerus, there is a mass-like, long T1 mixed T2 signal with clear boundaries, measuring approximately 5.4 cm × 2.7 cm × 2.6 cm. On DWI, parts of the lesion show high signal, with surrounding patchy low signals. The adjacent bone is thinned, and there is a suggestion of discontinuity in the local bone, with uneven signal intensity, slightly reduced compared to previous scans, with good alignment in the upper segment. Surrounding soft tissue swelling is noted, which has significantly improved compared to before, with a reduction in solid components on enhanced scanning and marked enhancement.

**Fig. 6:**
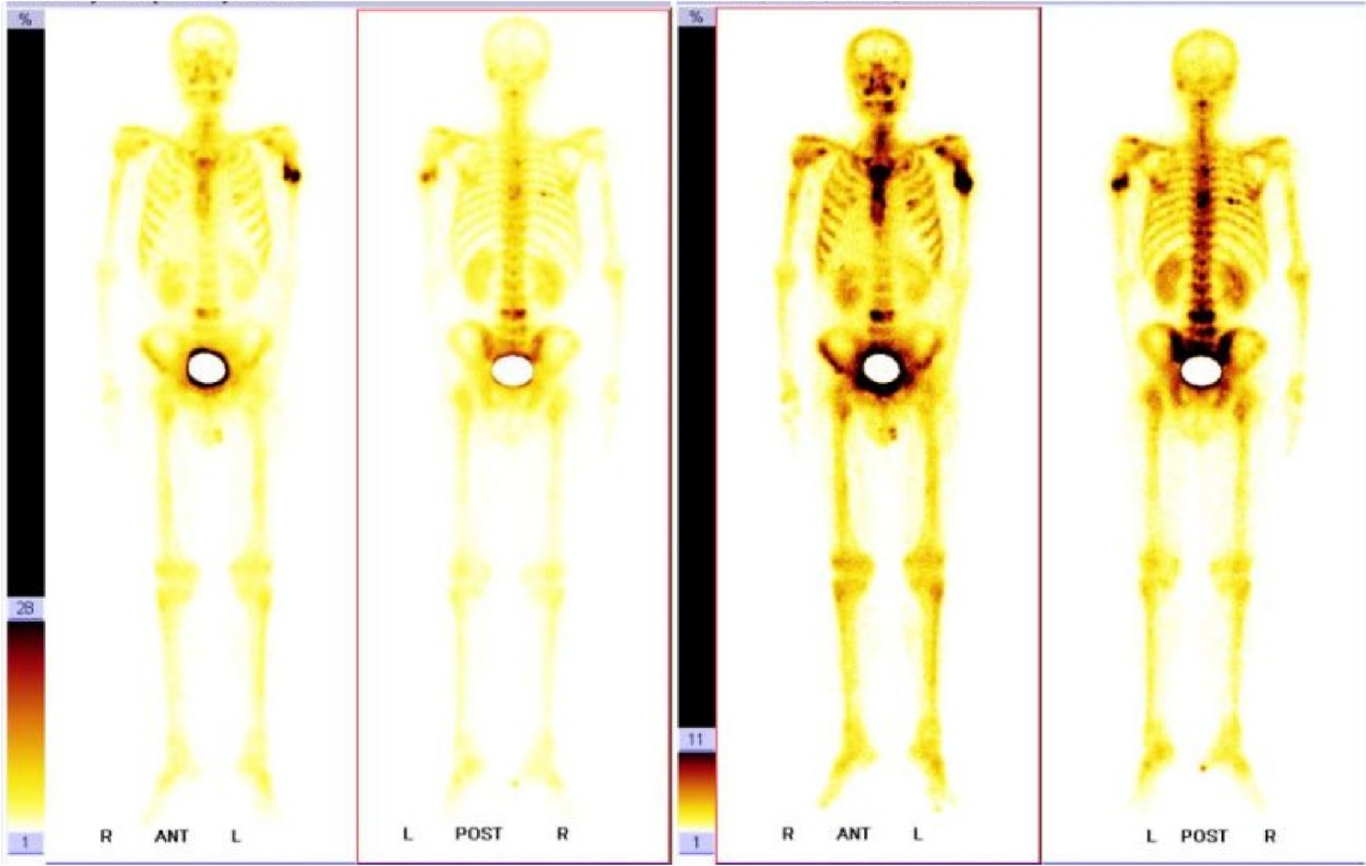
Seven months after treatment, follow-up reveals radioactive uptake in the left humerus, multiple vertebrae, and ribs

Seven months later, there was radiotracer uptake in the left humerus, cervical vertebrae, thoracic vertebrae, lumbar vertebrae, and multiple ribs (Fig. 6). This was considered to be multiple bone metastases from a malignant tumor.

## Discussion

DFSP encompasses various histopathological subtypes, including the classic type, fibrosarcomatous type, myxoid type, and pigmented type, among which the classic type is the most common. The classic type is characterized histologically by spindle-shaped tumor cells arranged in a storiform or cartwheel pattern, often infiltrating the subcutaneous fat tissue to form a honeycomb-like structure. The fibrosarcomatous type features tumor cells arranged in a herringbone or fishbone pattern, which confers greater invasiveness and a higher risk of distant metastasis [6]. In this paper, both the primary popliteal lesion and the metastatic mass in the humerus show spindle cells arranged in a herringbone pattern under light microscopy. In immunohistochemistry, both show strong positivity for Vimentin. However, individual test indicators such as CD34 are not completely consistent. This is considered to be caused by the heterogeneity of the tumor [7].

The pathogenesis of this disease remains unclear. Genetic studies have revealed that DFSP patients exhibit a (t 17;22)(q22;q13.1) chromosomal translocation or a fusion of the 17q22 and 22q13 loci into a ring chromosome. The resulting COL1A1/PDGFB fusion gene is considered the sole, specific genetic alteration in DFSP [8].

The European consensus interdisciplinary guidelines have staged dermatofibrosarcoma protuberans as follows: Stage I, non-elevated lesions, including atrophic or indurated plaques, macules, or small nodules; Stage II, elevated lesions; Stage III, lymph node metastasis; Stage IV, distant metastasis to other organs [9].The treatment of choice for DFSP is surgical resection, which is applicable to stages I, II, and even III and IV, typically including conventional excision, R0 resection, Mohs micrographic surgery (MMS), and partial/total amputation. Studies have shown that a margin of ≥ 3 cm significantly outperforms a margin of < 3 cm in reducing local recurrence rates. Additionally, radiotherapy can be used as an adjuvant or salvage treatment after multidisciplinary team assessment. Regarding the dosage of radiotherapy, 60 Gy should be given for uncertain or microscopically positive margins, and up to 70 Gy should be given for grossly positive margins or primary gross tumors. Whenever feasible, the radiation field should extend 3–5 centimeters beyond the surgical margins or primary tumor boundaries. Individual doses can be 2 Gy per day, five times a week [10, 11]. In this case, the biopsy of the patient’s left humerus lesion confirmed it to be a distant metastasis of DFSP.DFSP is usually diagnosed through incisional or, less often, excisional biopsy combined with anatomopathological tests. Fine-needle aspiration biopsies are rarely used due to their low accuracy. In this case, an incisional biopsy was chosen to confirm the pathology while minimizing patient harm [7].Taking into account the location and size of the bone metastasis, the characteristics of fibrosarcomatous DFSP, the patient’s physical condition, and the requirements for future quality of life, the remote consultation experts suggested that amputation surgery was not advisable. Instead, they recommended starting imatinib as an adjuvant therapy before reassessing the surgical option, followed by continued adjuvant imatinib treatment after surgery. The patient chose conservative treatment and was re-evaluated after two months of imatinib therapy, showing a slight reduction in the tumor size. Unfortunately, at the seven - month follow - up, the humeral lesion had progressed, and, in addition, multiple bone metastases were found in the cervical spine, thoracic spine, and bilateral ribs. This case also reminds us that DFSP is highly invasive. Aggressive and comprehensive treatment, along with regular follow - up, is of great importance. In my opinion, it was the insufficient awareness of DFSP that led to the delay in detecting the metastatic tumor in the proximal humerus, which had reached a length of 6.2 cm only one and a half years after the excision of the popliteal fossa lesion. We hope to use this case to raise awareness about the high invasiveness and concealment of DFSP, and to emphasize the need for early wide excision and regular follow - up to rule out distant metastasis.

Skeletal metastasis is more commonly associated with carcinomas, accounting for 85%−90% of cases, while sarcomas make up 10–15%. The predilection sites for skeletal metastasis are closely related to the hematopoietic function of the bone marrow, often occurring in areas of red marrow and predominantly concentrated in the bones of the trunk. Metastatic lesions rarely occur in the bones below the elbows. Metastatic DFSP is rare, with a generally accepted incidence of no more than 6%. The lower likelihood of metastasis may be related to the typically superficial nature of DFSP, its mostly low to intermediate pathological grade, and relatively small tumor size [5]. DFSP metastasizing to bone is exceedingly rare. Typically, the metastasis of tumor cells to the bones primarily depends on their intrinsic biological characteristics and their interaction with the bone microenvironment. Analyzing the causes of this disease from this perspective, we can consider the following aspects: On one hand, in terms of biological characteristics, the tumor cells of fibrosarcomatous DFSP exhibit significant atypia and frequent mitotic figures, possessing strong invasive capabilities, which provide the initial impetus for the tumor to metastasize to distant bones. On the other hand, in terms of the bone microenvironment, mesenchymal stromal cells in the bone marrow provide ample energy and nutrients for tumor cells that have metastasized there, promoting the trans-endothelial migration and proliferation of tumor cells within the bone marrow.

Tumors metastasize to bone primarily through direct invasion, hematogenous spread, and lymphatic dissemination. Regardless of the route by which the primary lesion metastasizes to the bone, it can result in osteolytic, osteoblastic, or mixed manifestations. It is generally believed that rapidly growing or highly vascularized tumors tend to exhibit osteolytic features, while osteoblastic changes are thought to result from tumor cells causing circulatory disturbances that stimulate bone formation [12]. This study hypothesizes that the mechanism of DFSP metastasizing to the humerus may involve hematogenous or lymphatic spread to the proximal medullary cavity of the humerus, with tumor growth progressing from the medullary cavity outward to the cortical bone and from the proximal to the distal end. The loose structure of the medullary cavity allows tumor cells that settle there to proliferate rapidly, but the hard bone soon restricts further expansion. Under the interaction of these two factors, the cortical bone becomes thin. As tumor cells continue to grow and divide, the cortical bone is compressed and thinned, leading to pathological fractures. Adjacent blood vessels and nerves are consequently damaged, causing pain or restricted movement, prompting the patient to seek medical attention.

The imaging manifestations of bone metastases are highly variable, reminding us of the importance of correlating imaging findings with clinical history. In this case, the lesion is confined to a single humerus, presenting as a solitary occurrence with a narrow transition zone to the normal bone and a clear, sharp boundary, suggesting a benign or low-grade malignant biological behavior. CT imaging indicates that the lesion has a relatively uniform density overall, consistent with soft tissue density, with thinning and localized discontinuity of the adjacent cortical bone, exhibiting expansile bone destruction. MRI, with its higher tissue resolution, reveals heterogeneous T2 signal intensity within the lesion, restricted diffusion, and surrounding patchy tissue edema. If one were to interpret the images in isolation, there might be a risk of misdiagnosing it as a primary condition, such as bone lymphoma or certain types of sarcoma.

Some articles suggest that malignant tumor cells present in the bone are the source of cells that ultimately metastasize to other organ systems [13]. This also indicates that the prognosis for this case is likely to be poor, and systemic conditions should be included in the follow-up scope during regular check-ups. Additionally, this case reminds us that when encountering patients presenting primarily with pain in a certain bone at the initial diagnosis, it is crucial to thoroughly inquire about their past medical history and surgical history, conduct a comprehensive analysis, and make an integrated judgment. If necessary, a biopsy should be performed first to determine the origin, providing patients with as comprehensive a range of treatment options as possible to enhance their quality of life.

## Conclusion

Metastasis of DFSP is rare, and metastasis to the bone is even rarer. We need to combine the medical history with immunohistochemical and molecular pathology monitoring as well as imaging examinations for a comprehensive diagnosis. It is recommended to regularly perform imaging examinations to monitor potential recurrence and metastasis.

## Data Availability

No datasets were generated or analysed during the current study.
